# Untying Surface Chemistry and Emulsion Stability to Construct Multifunctional Pickering Emulsion SERS Sensors for Pretreatment‐Free Quantitative Analysis in Bio‐Media

**DOI:** 10.1002/advs.202505714

**Published:** 2025-05-14

**Authors:** Yingrui Zhang, Chunchun Li, Ruairi Carland, Ziwei Ye, Steven E. J. Bell, Yikai Xu

**Affiliations:** ^1^ School of Materials Science and Engineering East China University of Science and Technology 130 Meilong Road Shanghai 200237 China; ^2^ Key Laboratory for Advanced Materials and Feringa Nobel Prize Scientist Joint Research Center Frontiers Science Center for Materiobiology and Dynamic Chemistry School of Chemistry and Molecular Engineering East China University of Science and Technology 130 Meilong Road Shanghai 200237 China; ^3^ School of Chemistry and Chemical Engineering Queen's University Belfast University Road Belfast BT7 1NN UK

**Keywords:** core‐shell, interface self‐assembly, internal standard, Pickering emulsion, SERS

## Abstract

Plasmonic Pickering emulsions have immense potential as enhancing substrates in surface‐enhanced Raman spectroscopy (SERS). Traditionally, the functional nanoparticles also act as the emulsion stabilizer, so that their surface chemistry is tied directly to emulsion stability. However, this has meant that adsorption of molecules to the plasmonic nanoparticles destabilizes the emulsion system, which severely limits the use of Pickering emulsions in SERS. Here, we used a dual‐particle approach to create plasmonic Pickering emulsions, in which emulsion stability is maintained solely by one type of nanoparticle so that the other could be used to provide functionality without constraints to its surface properties. This allowed us to construct multiwalled carbon nanotubes‐Au@Prussian blue Pickering emulsion SERS sensors with integrated internal standards and filtration functionalities, which enabled quantitative, biphasic and multiplex analysis of discrete molecules in serum. The synthetic approach used in this work can be readily extended to form Pickering emulsions carrying functional components with arbitrary surface functionalities, which paves the way for advanced applications in sustainability and healthcare.

## Graphical abstract

A dual‐particle interfacial self‐assembly approach is used to create surface‐accessible Pickering emulsions with Au@Prussian blue nanoparticles acting as the functional component. These emulsions have strong plasmonic properties and are equipped with molecular sieves and internal standards. This allows them to be used as miniature sensors for pretreatment‐free quantitative analysis of bio‐samples using surface‐enhanced Raman spectroscopy.

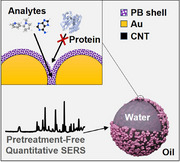

## 1 Introduction

Surface‐enhanced Raman spectroscopy (SERS) is a powerful and non‐destructive analytical technique that can be used to study molecules adsorbed near the surface of plasmonic enhancing materials with molecular specificity and single‐molecule level sensitivity.^[^ ^1^, ^2^, ^3^ ^]^ The nature of SERS means that the functionality and property of the enhancing substrate material will dictate the effectiveness and generality of the SERS approach. This is a double‐edge sword, which on one hand has made applications of SERS challenging, since the enhancing material need to simultaneously be structurally stable, plasmonically active and surface‐accessible.^[^ ^4^, ^5^, ^6^ ^]^ However, on the other hand, the unique requirement for an enhancing material in SERS also presents significant opportunities in designing tailored solutions for complex analytical problems through smart‐material design.^[^ ^7^, ^8^, ^9^, ^10^ ^]^ This makes the development of multifunctional enhancing substrates which enable direct identification and quantitative analysis of complex real‐life samples a holy‐grail in SERS, since this would pave the way for important applications such as bedside clinical studies and on‐site forensic analysis.^[^ ^11^, ^12^, ^13^, ^14^ ^]^

Pickering emulsions which are particle‐coated liquid droplets suspended in a second immiscible liquid phase are featured by their large surface area and bi‐phasic nature.^[^ ^15^, ^16^ ^]^ These exciting and unique characteristics make Pickering emulsions formed via plasmonic nanoparticles highly anticipated multifunctional enhancing substrate materials for biphasic or interfacial SERS analysis. Fundamentally speaking the stabilization of Pickering emulsions requires the nanoparticle stabilizers to maintain a particular surface hydrophobicity.^[^ ^17^ ^]^ For plasmonic Pickering emulsions, this has meant that the surface of the Ag or Au nanoparticles needed to be modified with particular types of ligands and that the adsorption of other types of functional or analyte molecules to the plasmonic nanoparticles’ surface would alter their hydrophobicity and destabilize the emulsion system.^[^ ^18^ ^]^ This problem becomes even more pronounced in the analysis of complex real‐life samples, which could contain various types of chemical species that adsorb to the nanoparticles and alter their surface hydrophobicity.^[^ ^19^ ^]^

The first report of plasmonic Pickering emulsions was made more than 30 years ago by Efrima et al, in which SERS was employed as a characterization technique to study the adsorbed modifier ligands on the surface of the plasmonic nanoparticles.^[^ ^20^ ^]^ However, as a result of the strong dependence of emulsion stability on particle surface chemistry, there have only been a handful of cases demonstrating the use of plasmonic Pickering emulsions in SERS since then.^[^ ^21^, ^22^, ^23^, ^24^, ^25^ ^]^ More specifically, Ling et al. synthesized plasmonic Pickering emulsions stabilized with perfluorodecanethiol‐modified Ag nanocubes, which allowed multiplex detection of methylene blue, rhodamine 6G and dimethyl yellow.^[^ ^21^ ^]^ Similarly, Li et al. synthesized plasmonic Pickering emulsions stabilized with perfluorodecanethiol‐modified Au nanostars, which allowed simultaneous quantification of methylation ratio and ^5m^C level in DNA.^[^ ^22^ ^]^ Tang et al. synthesized plasmonic Pickering emulsions stabilized with HS‐β‐cyclodextrin‐modified Ag nanoparticles.^[^ ^23^ ^]^ The HS‐β‐cyclodextrin not only acted as a modifier to adjust surface hydrophobicity of the Ag nanoparticles, but also as a supramolecular host to capture *o*‐phenylenediamine, which allowed SERS kinetics monitoring of the catalytic conversion of *o*‐phenylenediamine to either benzotriazole or 2,3‐diaminophenazine. The cases shown above were achieved by identifying analyte molecules which fortuitously interacted with the molecular modifier without significantly altering the surface hydrophobicity of the plasmonic nanoparticles. As a result, only a handful of analytes have been successfully detected. Beyond these rare examples, the use of Pickering emulsions in SERS have required them to be further converted into colloidosomes or polymeric hybrid materials which compromises the unique biphasic properties of the emulsions that is crucial for applications such as minute bioanalysis and dual‐phase sensing.^[^ ^26^, ^27^, ^28^, ^29^ ^]^

In this work we demonstrate the synthesis of a new class of multifunctional plasmonic Pickering emulsion‐based miniature SERS sensors which can be used for quantitative, biphasic and multiplex analysis of a wide range of target molecules, including neurotransmitters, drugs, illegal food additives, environmental contaminants and pesticides. This builds on our newly developed dual‐particle approach to create Pickering emulsions, where one type of particle (for example multiwall carbon nanotubes, MWCNTs) acted as the emulsion stabilizer which paved the way for surface functionalization of the other type of nanoparticle.^[^ ^18^ ^]^ In this work, we exploited this advantage by functionalizing the surface of the plasmonic Au nanoparticles with a Prussian blue (PB) shell to construct MWCNT‐Au@PB emulsions. Importantly, the Au core provided strong plasmonic enhancement while the porous PB shell acted as a molecular filter and internal standard. This enabled the MWCNT‐Au@PB Pickering emulsions to be used as miniature SERS sensors for precise and accurate quantitative analysis in complex sampling environments, such as serum, without the need for any sample pre‐treatment. More broadly, the synthetic approach used in this work can be readily extended to form Pickering emulsions carrying plasmonic functional components with arbitrary surface functionalities, which paves the way for the rational construction of biphasic miniature sensors tailored for advanced applications in SERS and beyond.^[^ ^30^ ^]^

## 2 Results

### 2.1 Synthesis of Au@PB Colloid

**Figure 1:**
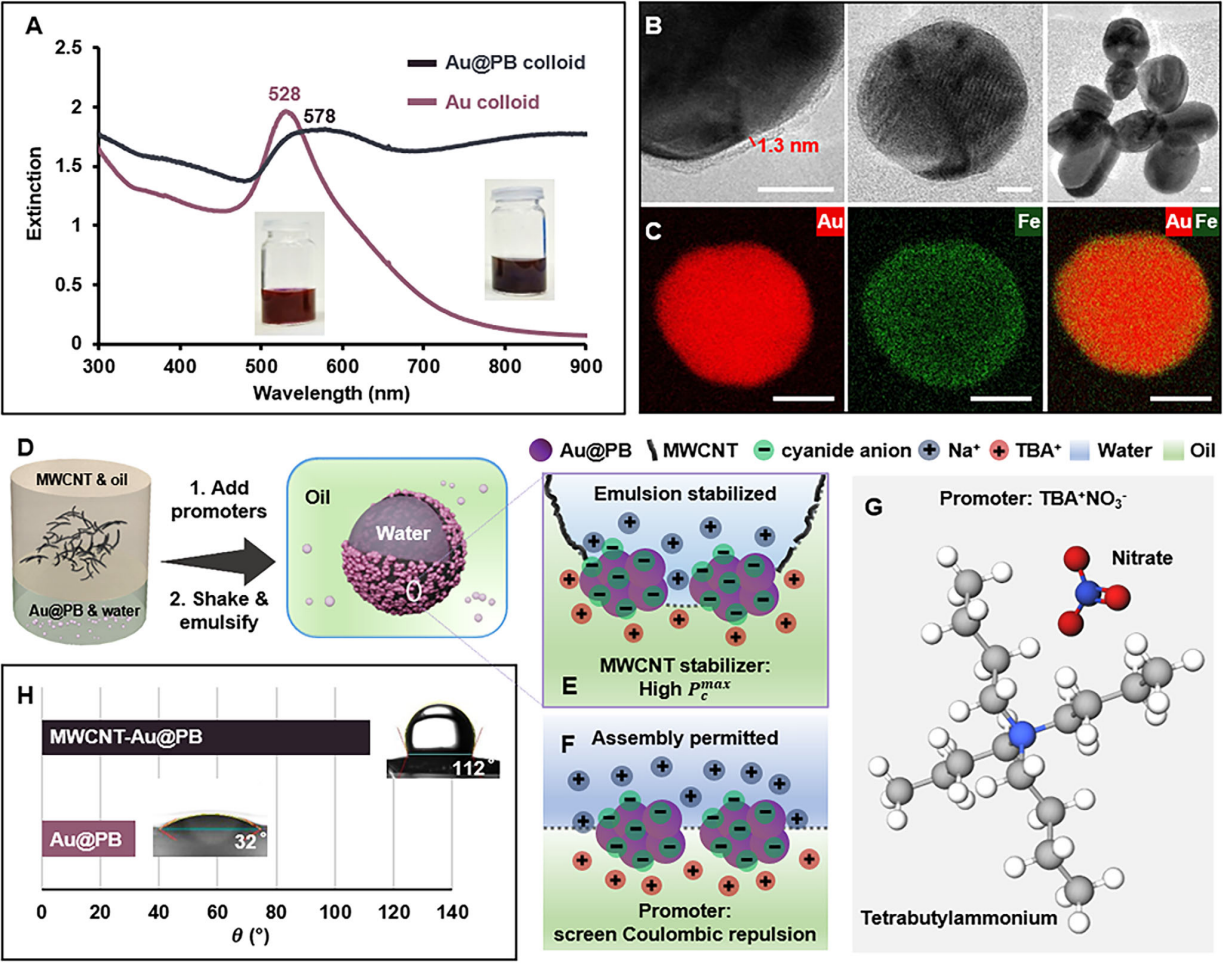
Au@PB nanoparticles and their assembly into Pickering emulsions. (A) UV‐vis spectra of Au and Au@PB colloid. (B) HRTEM showing the PB shell layer and typical Au@PB nanoparticles and agglomerates. (C) HAADF‐EDX analysis of a typical Au@PB nanoparticle. The scale bars in (B‐C) correspond to 10 and 20 nm, respectively. (D) Schematic illustrations of the synthetic procedure of MWCNT‐Au@PB Pickering emulsions. (E) Schematic illustrations of the MWCNT stabilizer increasing the maximum capillary pressure that could be withstood between emulsion droplets. (F) Schematic illustration of the promoter acting as a charge‐screening agent in interfacial self‐assembly. (G) The molecular structure of the amphiphilic TBA^+^ NO_3_^−^ promoter. (H) Three‐phase contact angle measurements of Au@PB and MWCNT‐Au@PB in water‐cyclohexane.

We start off with the synthesis of Au@PB nanoparticles. The colloid was synthesized using a previously reported double‐precursor method which involved etching of the surface of citrate‐stabilized Au colloidal nanoparticles by CN^−^, followed by in‐situ growth of a PB shell on the Au surface using Fe^3+^ and Fe^2+^ salts (Figure S1 and Methods).^[^ ^31^ ^]^ As shown in **Figure** **1**A, this led to the formation of a stable colloidal dispersion of Au@PB nanoparticles which resembled that of the parent Au colloid but with a slightly darkened colour. This was reflected in the extinction spectrum of the Au@PB colloid which was red‐shifted by ca. 50 nm compared to the Au colloid and had a broadened extinction band. Further ex‐situ analysis of the Au and Au@PB colloids using transmission electron microscopy (TEM) showed that the Au@PB colloid was partially agglomerated and consisted of a mixture of single Au@PB nanoparticles as well as agglomerates composed of a few to tens of individual nanoparticles (Figure 1B and Figure S2), which is consistent with the broadening of the extinction peak for Au@PB samples observed using UV‐vis spectroscopy. The structure of the PB shell was studied using high resolution TEM (HRTEM) and high‐angle annular dark field‐scanning transmission electron microscopy‐energy‐dispersive X‐ray spectroscopy (HAADF‐STEM‐EDX). As shown in Figure 1B, the PB shell was ca. 1.3 nm thick, which was ideal for generating a strong plasmonic response and to act as a molecular filter in SERS applications.^[^ ^32^, ^33^ ^]^ In addition, the EDX results showed that the PB shell was coated onto the surface of each Au nanoparticle even in the agglomerates (Figure 1C and Figure S3), which is important in ensuring the functionality of the Au@PB nanoparticles in further SERS applications as will be demonstrated below.

### 2.2 Synthesis of MWCNT‐Au@PB Plasmonic Pickering Emulsions

The formation of plasmonic Pickering emulsions is illustrated in Figure 1D, which in practice, simply involves shaking an oil solution containing the hydrophobic MWCNT stabilizers with the aqueous Au@PB colloid containing micromolar concentrations of promoter ions. Microscopically, the formation of stable Pickering emulsions involves two steps, (i) the migration of nanoparticles to the water‐oil interface and (ii) the stabilization of liquid emulsion droplets from coalescence. It is now well‐established that the adsorption of solid nanoparticles to the interface between two immiscible fluids is an energetically favourable process that lowers interfacial energy.^[^ ^34^ ^]^ This is shown in Equation (1) which specifically describes the loss in interfacial energy for the migration of a spherical nanoparticle to the interface within the water‐in‐oil emulsions that are relevant to this work,

(1) $$\Delta G=-\pi R^{2}\gamma_{w-o}{\left(1-\cos\theta \right)}^{2}$$

where *R* is the radius of the nanoparticle, γ_w − o_ is the surface tension between water and oil, θ is the three‐phase contact angle measured through the dispersed phase on a solid particle in the environment of the dispersion medium for water‐in‐oil emulsions. It should be noted that while this equation is derived using spherical nanoparticles as an example, it shows that the loss in interfacial energy is positively correlated to the amount of interfacial area removed by inserting a nanoparticle to the interface. This means that the interfacial self‐assembly method is extremely versatile and can be used to assemble nanoparticles with varying geometries.^[^ ^35^ ^]^

However, for aqueous colloids which are commonly stabilized via interparticle Coulombic repulsion, an additional electrostatic energy barrier needs to be overcome in order for the nanoparticles to assemble into tightly packed arrays at the water‐oil interface,^[^ ^36^ ^]^ which is particularly important for the generation of plasmonic hot‐spots in SERS. Indeed, the Au@PB nanoparticles, which had a measured surface zeta potential of ca. ‐35 mV, did not assemble spontaneously at the water‐oil interface as observed in our experiments. The conventional approach to overcome interparticle electrostatic repulsion in the synthesis of plasmonic Pickering emulsions is to modify the surface of the charged‐nanoparticles with a charge‐neutral thiol modifier, such as the HS‐β‐cyclodextrin and perfluorodecanethiol modifiers mentioned above.^[^ ^21^, ^23^ ^]^ However, modifying the surface of the plasmonic nanoparticles with strongly adsorbed capping ligands limits the adsorption of analyte molecules which is undesirable for SERS applications.^[^ ^37^ ^]^ Therefore, in order to overcome interparticle electrostatic repulsion while maintaining the surface‐accessibility of the plasmonic nanoparticles, we used tetrabutylammonium nitrate (TBA^+^ NO_3_^−^) to promote the assembly of Au@PB nanoparticles at the water‐oil interface via a charge‐screening effect.^[^ ^38^, ^39^ ^]^ As shown in Figure 1F,G, the TBA^+^ is an organo‐electrolyte which migrated in a charged form into the oil phase. This unique amphiphilic property allowed the positively charged TBA^+^ ions to assemble alongside the negatively charged Au@PB nanoparticles at the water‐oil interface to generate densely packed nanoparticle assemblies.

In order to form stable Pickering emulsions, the nanoparticles assembled at the water‐oil interface must also be able to prevent the liquid emulsion droplets from coalescence. This can be viewed as the problem of increasing the maximum capillary pressure ($P_{c}^{\max}$) that can be withstood by the bilayer of nanoparticles between two adjacent emulsion droplets, as shown in Figure 1E. $P_{c}^{\max}$ can be described by Equation (2) with the emulsion system becoming more stable at higher $P_{c}^{\max}$ values,^[^ ^40^ ^]^

(2) $$P_{c}^{\max}=\pm p\frac{2\gamma_{w-o}}{R}\left(\cos\theta \pm z\right)$$

where *p* and *z* are systematic parameters provided in Supplementary Table S1. In Equation (2), the sign “± ” becomes “+ ” for oil‐in‐water emulsions, and “− ” for water‐in‐oil emulsions. Equation (2) shows that the value of $P_{c}^{\max}$ is directly related to the θ of the nanoparticles at the water‐oil interface, which was measured to be 32° for pure Au@PB nanoparticles and 112° for a mixture of MWCNT‐Au@PB nanoparticles, as shown in Figure 1H. From the measured θ values it can be deduced that the hydrophilic Au@PB nanoparticles could potentially stabilize oil‐in‐water emulsions while the hydrophobic MWCNT‐Au@PB nanoparticle mixture would be more suitable as stabilizers for water‐in‐oil emulsions. Therefore, substituting the experimental values of θ_Au@PB_ and θ_MWCNT − Au@PB_ into the appropriate forms of Equation (2), gave the $P_{c}^{\max}$ values for emulsions formed with just Au@PB or a mixture of MWCNT‐Au@PB nanoparticles, as shown in Equation.^[^ ^3^, ^4^ ^]^

(3) $$P_{c}^{\max}\left(\mathrm{Au}@\mathrm{PB}\right)\approx 5.15\frac{\gamma_{w-o}}{R_{\mathrm{Au}@\mathrm{PB}}}$$

(4) $$P_{c}^{\max}\left(\mathrm{MWCNT}-\mathrm{Au}@\mathrm{PB}\right)\approx 6.66\frac{\gamma_{w-o}}{R_{\mathrm{MWCNT}-\mathrm{Au}@\mathrm{PB}}}$$

It should be noted that the calculations above were performed under the approximation that the particles were spherical, but in fact it has been shown that the wire‐like structure of MWCNTs allowed them to cover and stabilize significantly larger interface area compared to spherical structures.^[^ ^41^ ^]^ This would be reflected as the actual *p* and *z* values becoming higher compared to the approximated values used in Equation (4), since both of these systematic values are positively correlated to the particle coverage at the interface. More generally, the calculations above predicted that the MWCNT‐Au@PB Pickering emulsions would be significantly more resistant to coalescence compared to Au@PB Pickering emulsions. This was consistent with literature and our experimental observations which showed that Au@PB nanoparticles alone generated unstable emulsions which coalesced into a bulk 2‐dimensional interfacial assembly within minutes while MWCNT‐Au@PB Pickering emulsions remained stable for more than a month, as shown in Figure S4. As a comparison, in cases when Au@PB nanoparticles was co‐assembled with SiO_2_ nanoparticles which were very hydrophilic due to the presence of a large number of hydroxyl groups on their surface, oil‐in‐water type Pickering emulsions can be formed which also remained stable for days and possessed strong SERS activity (Figure S5).

**Figure 2:**
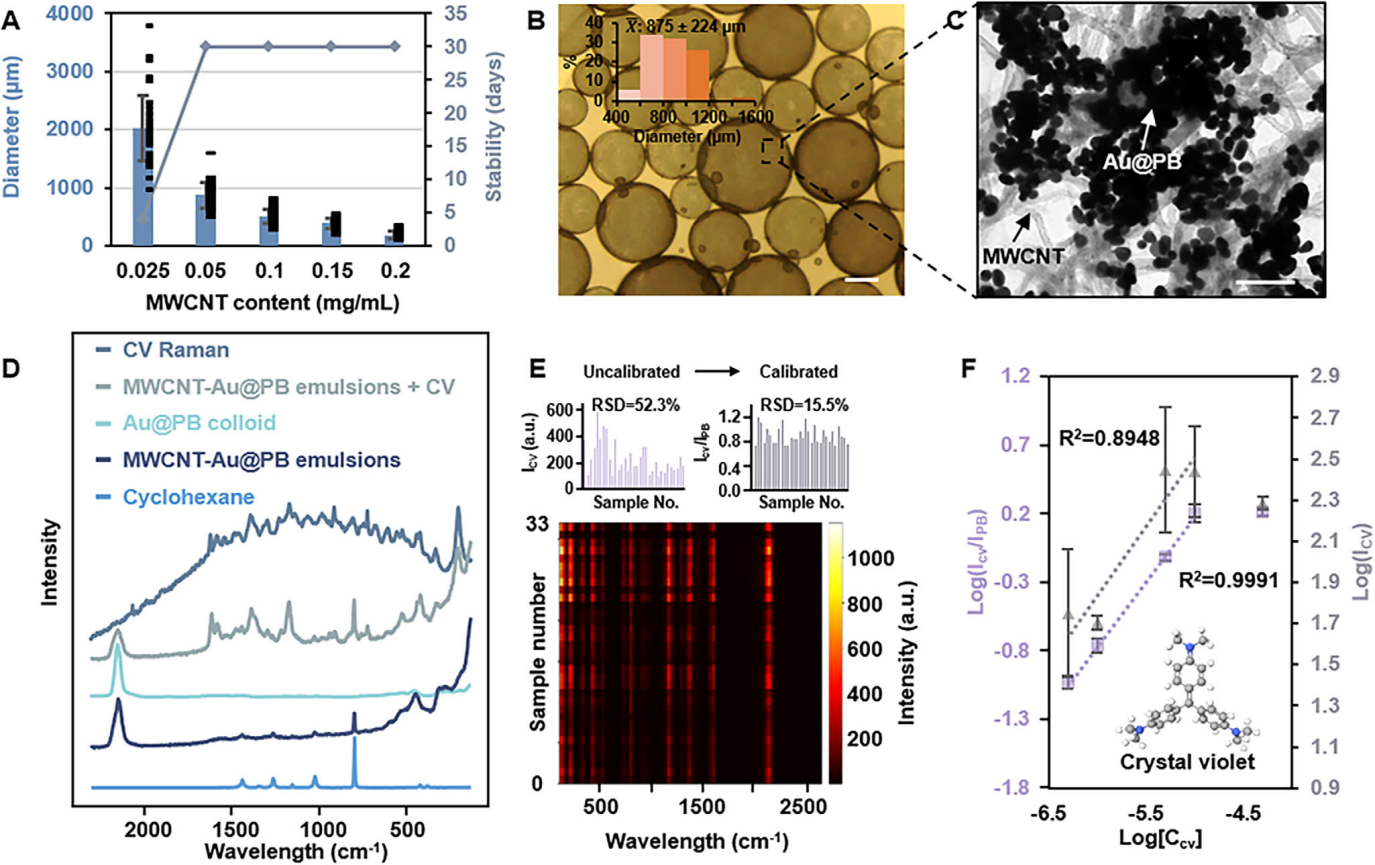
Material properties of MWCNT‐Au@PB Pickering emulsions. (A) Plot showing the average diameter and stability of the emulsion droplets in relation to the amount of MWCNT stabilizer used for emulsion formation. (B) A typical sample of MWCNT‐Au@PB Pickering emulsions formed with 0.05 mg/mL of MWCNT observed using an optical microscope. The scale bar corresponds to 500 µm. (C) A typical area of the nanoparticle layer on a MWCNT‐Au@PB Pickering emulsion droplet formed with 0.05 mg/mL of MWCNT observed using TEM. The scale bar corresponds to 200 nm. (D) SERS spectra obtained from Au@PB colloid, MWCNT‐Au@PB Pickering emulsions, MWCNT‐Au@PB Pickering emulsions spiked with a final concentration of 10^−5^ M of crystal violet (CV). Raman spectra of 10^−2^ M CV and pure cyclohexane. (E‐F) The SERS signal intensity of CV measured on a total of 33 random points using MWCNT‐Au@PB Pickering emulsions with or without using PB as an internal for signal calibration. The peak at 1172 and 2153 cm^−1^ was measured for CV and PB, respectively. The final concentration of CV in the emulsion sample was 10^−5^ M. (F) Linear calibration curve obtained by plotting Log(I_CV_) or Log(I_CV_/I_PB_) against Log[C_CV_] obtained. The data points in (F) were calculated from 4 independent experiments.

Since the MWCNTs acted as the stabilizer for the Pickering emulsions, this meant that it was possible to fine tune the properties of the emulsions by altering the amount of MWCNTs used. For example, it was found that the amount of MWCNTs present in the emulsion system directly impacted the average diameter of the Pickering emulsion droplets. For example, it was found that the amount of MWCNTs present in the emulsion system directly impacted the average diameter of the Pickering emulsion droplets. More specifically, increasing the amount of MWCNTs used allowed a larger interface area to be stabilized which led to smaller emulsion droplets, as shown in **Figure** **2**A and Figure S6. For the same reason, it would be expected that the size of the emulsion droplets is negatively correlated with the length of MWCNTs. This is potentially useful in applications of the Pickering emulsions as miniature SERS sensors, since this allowed the volume of each sensor droplet to be rationally controlled. Figure 2A also shows the size distribution and stability of the Pickering emulsions in regards to the concentration of the MWCNT stabilizers. From the data it could be seen that the emulsions were highly repeatable and remained highly stable (defined as the average diameter of the emulsion droplets changing by less than 10%, see Figure S7) for >30 days when the concentration of the MWCNT stabilizers were ≥0.05 mg/mL. A sudden and significant drop in the stability of the emulsions was observed when the concentration of the MWCNT stabilizers decreased to 0.025 mg/mL, which led to the formation of Pickering emulsion samples that were stable for ca. 5 days.

Microscopy analysis was performed to investigate the structure of the nanoparticle layer at the water‐oil interface of the MWCNT‐Au@PB Pickering emulsions. Figure 2B,C shows an optical image of a typical batch of MWCNT‐Au@PB Pickering emulsion sample formed with 0.05 mg/mL of MWCNT stabilizers, along with the TEM image of a typical area of the interfacial nanoparticle layer on an emulsion droplet. As expected, the interfacial nanoparticle layer consisted of both MWCNT stabilizers and Au@PB functional nanoparticles. The MWCNT stabilizers were randomly scattered in the form of a mesh while the Au@PB nanoparticles formed islands that provided strong plasmonic enhancement. Further SEM analysis of Pickering emulsions formed with different MWCNT concentrations revealed that the proportion of MWCNT stabilizers in the interfacial nanoparticle layer decreased with the concentration of MWCNTs used for emulsion synthesis (Figure S8). This was consistent with the trend observed for the stability of the emulsions in relation to the concentration of MWCNTs and explained the sudden drop in emulsion stability at 0.025 mg/mL MWCNT concentration, since at this concentration the interface was covered predominantly by the Au@PB nanoparticles.

### 2.3 SERS Performance of MWCNT‐Au@PB Pickering Emulsions

In general, the MWCNT‐Au@PB Pickering emulsions exhibited strong SERS activity for all the MWCNT concentrations tested. The SERS activity of the MWCNT‐Au@PB emulsions was found to be inversely related to the concentrations of MWCNT stabilizers, as shown in Figure S9. This was unsurprising since decreasing concentrations of MWCNT stabilizers meant a higher percentage of the plasmonic Au@PB nanoparticles in the interfacial nanoparticle layer, as shown in Figure S8. Therefore, further SERS studies were conducted using Pickering emulsions formed with 0.05 mg/mL MWCNT stabilizers to balance emulsion stability and plasmonic activity.

The SERS signature of a typical batch of MWCNT‐Au@PB Pickering emulsions is shown in Figure 2D. Since the MWCNT‐Au@PB Pickering emulsions were constructed without the use of modifiers, the SERS signature of the emulsions resembled that of the parent Au@PB colloid and was dominated by two vibrational bands at 451 cm^−1^ and 2153 cm^−1^, which can be attributed to the Fe‐C stretching vibrations and the C≡N vibration from the PB skeleton, respectively.^[^ ^42^ ^]^ The Raman vibrations for Au‐Cl and citrate were not observed, which showed that the original chloride and citrate ions adsorbed on the surface of the Au nanoparticles were displaced suggesting the formation of a complete PB shell. Several additional weak vibrational bands could be observed between 800–1443 cm^−1^ in the SERS signature of the Pickering emulsions. These could be attributed to the Raman signals of the cyclohexane oil phase. In contrast, the Raman signals of the MWCNTs were not observed presumably due to the amount of MWCNT being too low to generate detectable Raman signals under the current experimental conditions.

The SERS performance of the MWCNT‐Au@PB Pickering emulsions was tested using crystal violet (CV) as the model analyte, which is a bulky dye molecule (see Figure S10 for calculated molecular diameter of CV). As shown in Figure 2D, strong SERS signals of CV could be obtained using MWCNT‐Au@PB Pickering emulsions as the enhancing substrate, which showed that the porosity of the PB shell allowed access of the analyte molecules to the plasmonic hot‐spots that are located within interparticle junctions. Figure 2E, shows the SERS signal intensity of 10^−5^ M of CV measured using 33 different emulsion droplets from 3 different batches of emulsions, which showed large intensity fluctuations (RSD = 52.3%). However, this was not a problem since the SERS signals of the PB shell could act as an internal standard to calibrate intensity variations that arose from human error or any uneven distribution of plasmonic hot‐spots. As a result, the RSD was significantly decreased (RSD = 15.5%) when the PB vibration band at 2153 cm^−1^ was used as the internal standard to correct for the signal fluctuation between each SERS measurement. Between batches the RSD was only 12.6% after correction (Figure S11). The effect of the PB internal standard is even more prominent in the establishment of a calibration curve for quantitation. As shown in Figure 2F, without calibration the accuracy and precision of the measurements were not sufficient to allow quantitation with confidence, but when the data was calibrated with the PB internal standard a highly accurate and precise linear calibration curve (R^2^ = 0.999) could be obtained. On the other hand, since the stability of MWCNT‐Au@PB Pickering emulsions were provided by MWCNTs, the adsorption of CV molecules on Au@PB surface did not perturb the emulsion systems. This was evidenced by monitoring the size distribution of emulsion droplets and intensity of the CV signals which showed negligible change of both even after being left for one week (Figure S12). Given their excellent stability, MWCNT‐Au@PB Pickering emulsions can be used for quantitative SERS analysis which we show below in the following sections.

### 2.4 A General Biphasic SERS Sensor

**Figure 3:**
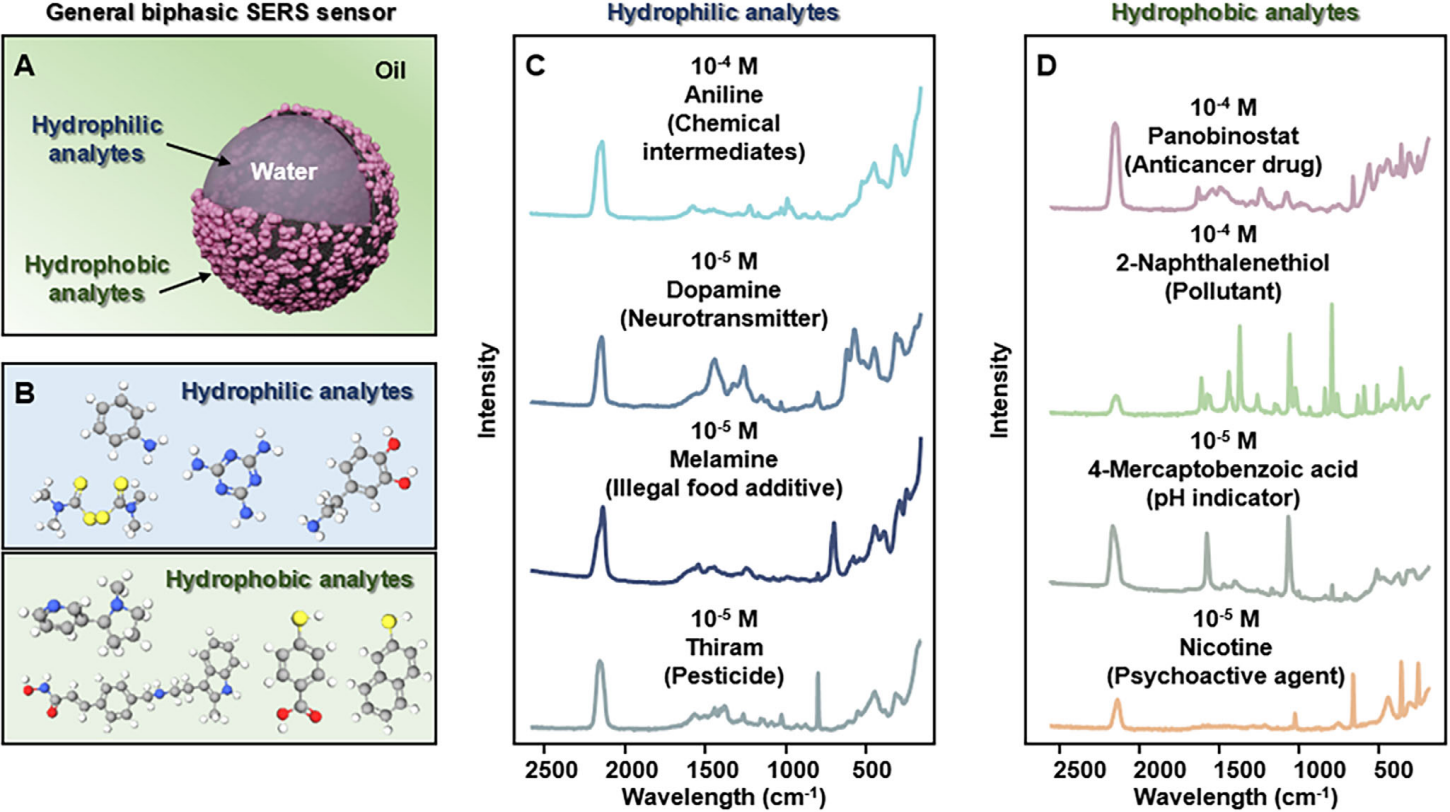
Biphasic SERS analysis. (A) Schematic illustration of the MWCNT‐Au@PB Pickering emulsions acting as a biphasic SERS sensor. (B‐D) The molecular structures of the hydrophilic and hydrophobic analytes and their correspond SERS spectrum obtained using MWCNT‐Au@PB Pickering emulsions.

Since the stability of the MWCNT‐Au@PB Pickering emulsions were not dependent on the surface chemistry of the plasmonic nanoparticles and that the plasmonic nanoparticles were not modified with strongly adsorbed modifiers, this unlocked the potential of the plasmonic Pickering emulsions as a generally applicable biphasic SERS sensor (**Figure** **3**A). As shown in Figure 3B, this was demonstrated using a wide range of analyte molecules with diverse chemical structures including thiol, amine, disulfide, pyridine and indoline. It should be noted that none of these analyte molecules had been previously detected using SERS with Pickering emulsions (see Table S2 for details), since they could not displace the modifier molecules and/or led to the destabilization of the emulsions if adsorbed.

More specifically, Figure 3C showed the SERS spectra of hydrophilic analyte molecules which were introduced from the aqueous phase of the emulsion system. These analyte molecules included thiram, dopamine, melamine and aniline, which represented pesticides, neurotransmitters, illegal food additives and chemical intermediates, respectively. In addition, Figure 3D showed the SERS spectra of hydrophobic analyte molecules which were introduced from the oil phase of the emulsion system. These analyte molecules included 4‐mercaptobenzoic acid, 2‐naphthalene thiol, nicotine and Panobinostat, which represented pH indicators, pollutants, psychoactive agents and anticancer drugs, respectively. Moreover, since our synthetic approach is generally applicable for emulsions formed with different oils, this meant that the properties of the oil phase could be tailored to the analyte molecule to improve solubility and avoid overlapping of the SERS signals. For example, the SERS of 4‐mercaptobenzioc acid and 2‐napthalene thiol were acquired using cyclohexane as the oil phase while SERS signals of nicotine and Panobinostat were acquired using chloroform as the oil phase (see Figure S13 for the Raman signature of cyclohexane and chloroform).

### 2.5 Multifunctional SERS Sensors for Direct Quantitation in Serum

A well‐known challenge in SERS analysis is the direct quantitation of analyte molecules in serum, since the sample matrix contains a high concentration of proteins that adsorb spontaneously to Ag and Au nanoparticles, which disrupts the structural stability and surface accessibility of the enhancing substrate.^[^ ^43^ ^]^ For this reason, up to now the SERS studies performed using Pickering emulsions have been limited to model samples of analyte molecules dispersed in pure solvents, since the protein molecules would displace the modifiers and destabilize the emulsions.

**Figure 4:**
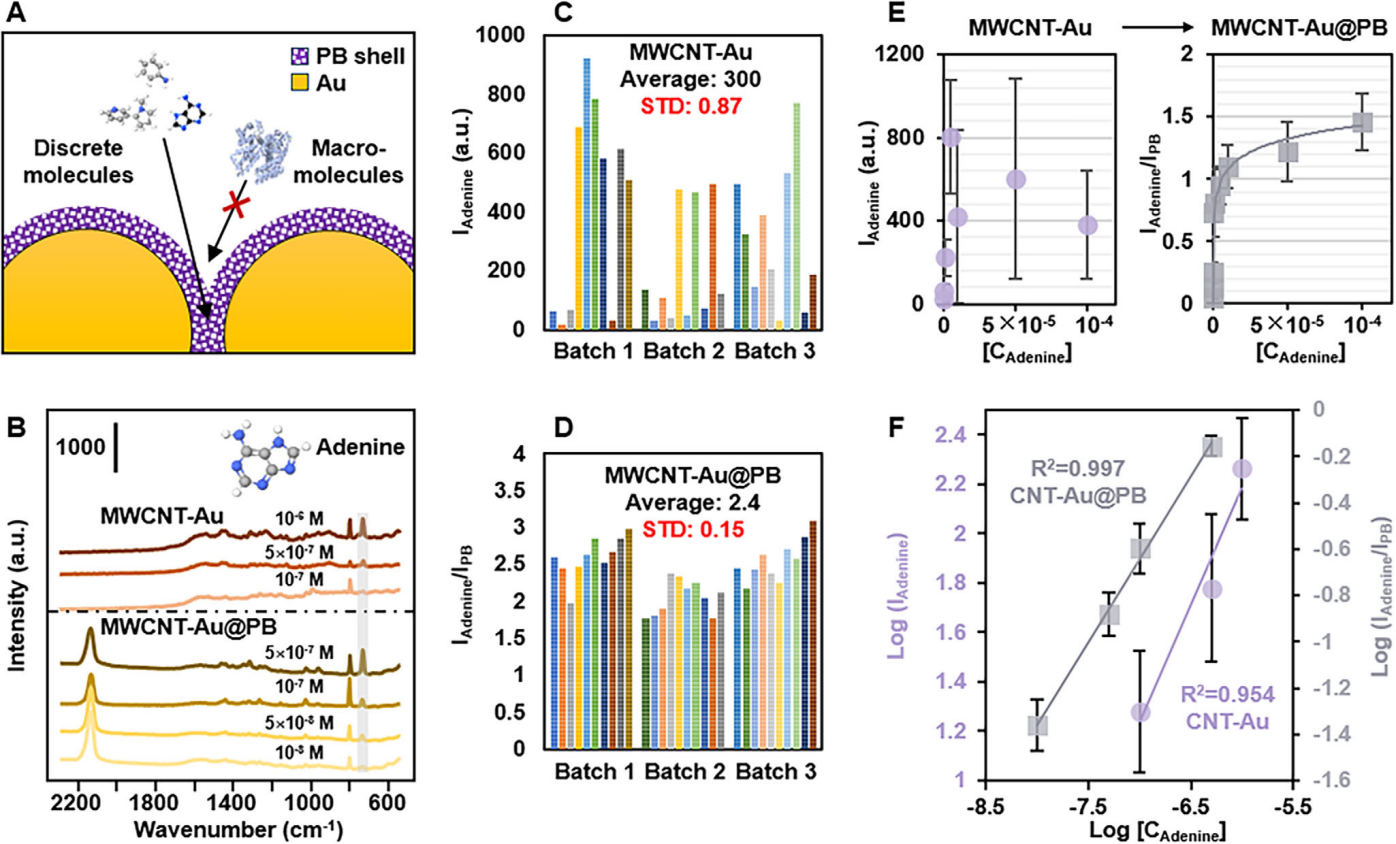
Quantitative SERS analysis in serum. (A) Schematic illustrations of the PB shell acting as a molecular filter allowing only the adsorption of discrete analyte molecules into the interparticle plasmonic hot‐spots. (B) Spectra set comparing the SERS signals of adenine in artificial serum obtained using MWCNT‐Au Pickering emulsions and MWCNT‐Au@PB Pickering emulsions. (C‐D) The SERS signal intensity of adenine in artificial serum measured on a total of 30 random points using MWCNT‐Au Pickering emulsions. The measured SERS signal intensity of adenine in artificial serum calibrated against the SERS signal intensity of the PB internal standard obtained from 30 random points using MWCNT‐Au@PB Pickering emulsions. The peak at 732 cm^−1^ was measured. The final concentration of adenine in the emulsion sample was 10^−5^ M. (E) I_adenine_ or I_adenine_/I_PB_ plotted against [C_adenine_] obtained using MWCNT‐Au and MWCNT‐Au@PB Pickering emulsions, respectively. (F) Linear calibration curve obtained by plotting LogI_adenine_ or LogI_adenine_/I_PB_ against Log[C_adenine_] obtained using MWCNT‐Au and MWCNT‐Au@PB Pickering emulsions, respectively. The data points in (E‐F) were calculated from 4 independent experiments.

The fact that the stability of the Pickering emulsions is no longer tied to the surface chemistry of the plasmonic Au nanoparticles meant that it was possible to tailor the surface properties of our Pickering emulsion SERS sensor to achieve direct quantitation in serum. More specifically, since the MWCNT stabilizers are chemically inert, this allowed the emulsions to remain highly stable in SERS studies even when artificial serum samples were added directly to the emulsions. Moreover, as shown in **Figure** **4**A, the presence of the porous PB shell over the plasmonic Au nanoparticles could act as a molecular filter which filtered out the macro‐proteins while allowing discrete molecules to access the plasmonic hot‐spots.^[^ ^44^, ^45^ ^]^ This effect is demonstrated in Figure 4B which showed direct SERS detection of adenine in serum with the limit of detection reaching 10^−8^ M. For comparison, we also synthesized MWCNT‐Au Pickering emulsions using the same self‐assembly approach. Although the SERS signals of adenine in artificial serum could be observed at higher concentrations using MWCNT‐Au Pickering emulsions, it decreased rapidly and disappeared abruptly below 10^−7^ M. This significant contrast in the limit of detection is a result of the competitive adsorption between the protein and adenine molecules that took place only on the MWCNT‐Au Pickering emulsions, where the Au was not protected by a PB functional layer.

In addition to effecting sensitivity, the competing protein molecules also negatively affected the reproducibility of the SERS analysis. More specifically, Figure 4C,D and Figure S14 compared the SERS signal of 10^−5^ M of adenine in artificial serum obtained using 3 different batches of MWCNT‐Au and MWCNT‐Au@PB Pickering emulsions. As shown in Figure 4C, the SERS signals of adenine fluctuated greatly (RSD = 87%) on MWCNT‐Au Pickering emulsions, but a clear improvement of the RSD by 18% was observed with the addition of a PB shell that filtered away the protein molecules (Figure S14), which demonstrated the negative effect of the competing protein molecules on the reproducibility of the SERS measurement. The reproducibility of the SERS measurements could be further improved by calibrating the SERS signals of the adenine molecule against the PB internal standard, which led to a significant improvement of the RSD to 15%, as shown in Figure 4D.

The intensity of the SERS vibration band at 732 cm^−1^ plotted against the corresponding concentration of adenine in samples of artificial serum is shown in Figure 4E,F. From the calibration plots it could be seen that large signal fluctuations were present for all adenine concentrations, which prevented meaningful quantitative measurements using MWCNT‐Au Pickering emulsions. In contrast, functionalizing the plasmonic Au nanoparticles with a PB shell allowed highly reproducible quantitative SERS analysis to be performed. Besides adenine, the MWCNT‐Au@PB Pickering emulsion sensors can also be readily applied to the direct quantitation of various other types of important discrete bio‐molecules in artificial serum, which is demonstrated Figure S15 using dopamine and melamine.

### 2.6 Biphasic SERS Sensors for Multiplex Analysis in Complex Samples

**Figure 5:**
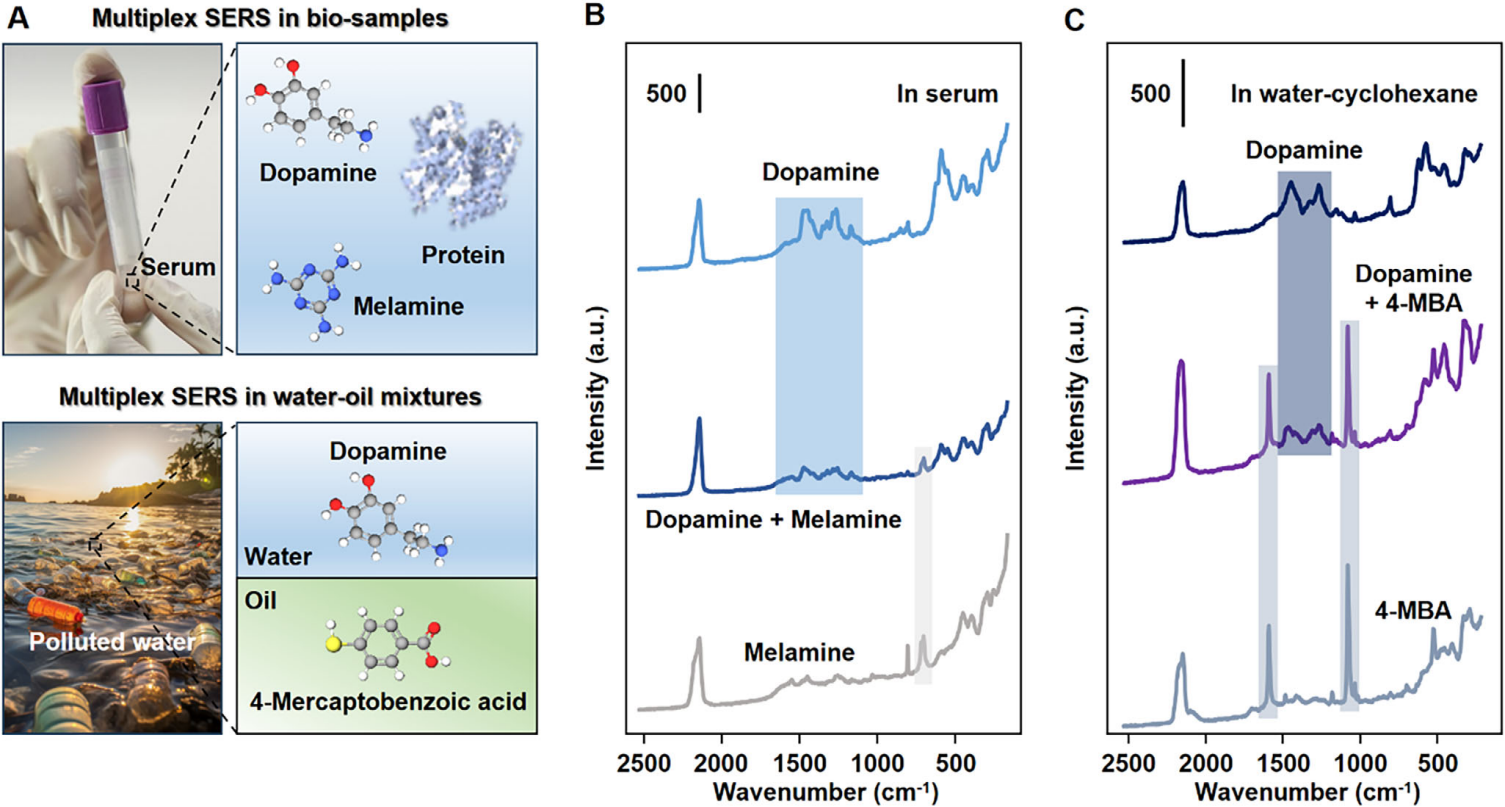
Multiplex SERS analysis in complex media. (A) Schematic illustrations demonstrating multiplex SERS analysis in bio‐solution and in water‐oil mixtures. (B) SERS spectra obtained from serum spiked with dopamine, melamine, a mixture of dopamine and melamine using MWCNT‐Au@PB Pickering emulsions. The final concentrations of the analytes were 10^−6^ M. (C) SERS spectra obtained water spiked with dopamine, cyclohexane spiked with 4‐mercaptobenzoic acid (4‐MBA), water‐oil mixtures spiked with dopamine and 4‐mercaptobenzoic acid using MWCNT‐Au@PB Pickering emulsions. The final concentrations of the analytes were 10^−5^ M.

In addition to the challenge brought about by the complexity of the sample matrix, another challenge in the analysis of real‐life samples is the need to be able to simultaneously detect and differentiate between multiple target molecules since this requires the analytical technique to be both widely applicable and molecularly specific.^[^ ^46^ ^]^ As shown above the MWCNT‐Au@PB plasmonic Pickering emulsions are versatile SERS substrates that satisfied all the requirements above. For example, **Figure** **5**B shows multiplex SERS analysis of two biologically relevant analyte molecules, melamine and dopamine in artificial serum. It could be seen that the SERS spectra consisted sharp vibrational bands from both melamine (703 cm^−1^) and dopamine (1474 cm^−1^), which allowed the two molecules to be simultaneously detected and identified according to their characteristic peaks. The example above was not a special case, another example where the MWCNT‐Au@PBs were used for multiplex SERS detection and identification of adenine and dopamine in horse serum is shown in Figure S16.

The examples above represented multiplex SERS analysis of aqueous samples that contained several types of discrete molecules of interest, which is typical for biological samples and foodstuff.^[^ ^47^, ^48^ ^]^ However, in environmental samples, such as oil contaminated wastewater, the sample may contain both hydrophilic and hydrophobic target molecules dissolved in a mixture of water and oil (Figure 5A).^[^ ^49^, ^50^ ^]^ This is not an issue when using the MWCNT‐Au@PB Pickering emulsions for SERS analysis since the biphasic property of the emulsions allows the enhancing nanoparticles to access analyte molecules dispersed in both the aqueous and oil phases. For example, Figure 5C shows biphasic multiplex SERS analysis of a hydrophobic analyte, 4‐mercaptobenzoic acid, and a hydrophilic analyte, dopamine. From the SERS spectra it can be seen that the characteristic peaks for 4‐mercaptobenzoic acid (1071 cm^−1^) and dopamine (1474 cm^−1^) can be clearly distinguished, which allowed the two types of analyte molecules to be easily detected and identified in the sample mixture.

## 3 Discussion

In summary, this work demonstrated a new approach for the construction of multifunctional plasmonic Pickering emulsions for SERS applications. Previous SERS analysis performed using Pickering emulsions have been limited to model systems due to the requirement for nanoparticle surface modification which prevented analyte adsorption. In contrast, our approach used MWCNTs as the emulsion stabilizer which allowed the plasmonic nanoparticles to be co‐assembled at the emulsion surface without the need for any surface modification. Importantly, this meant that the surface chemistry of the plasmonic nanoparticles were no longer tied to emulsion stability, which opened up their surface to the adsorption of analyte molecules and functional ligands alike. Using this approach we designed and constructed MWCNT‐Au@PB Pickering emulsions, in which the plasmonic Au nanoparticles were protected by a PB functional layer that acted as a molecular filter and SERS internal standard. We showed that the functionalities of the PB shell combined with the biphasic property of the emulsions paved the way for important but previously challenging SERS applications including direct quantitation and multiplex detection of discrete molecules in serum. Fundamentally speaking, the nature of our approach means that it can be used as a platform technique to rationally construct biphasic Pickering emulsion‐based SERS sensors with tailored plasmonic and surface functionalities. For example, we showed that the surface of the Au@PB nanoparticles can be adsorbed with 4‐mercaptobenzoic acid molecules, which are commonly employed as pH indicators in SERS; the Au@PB nanoparticles can be substituted with citrate‐capped Au nanoparticles, which are one of the most commonly used enhancing nanoparticles in SERS. More broadly, the ability to construct biphasic nanoparticle assemblies with fully customizable functionalities will benefit important applications spanning across sustainability and healthcare.

## 4 Experimental Section

### Materials

Multiwalled carbon nanotubes (MWCNTs), tetrachloroauric (III) acid trihydrate (HAuCl_4_·3H_2_O) (99.9999%), trisodium citrate dihydrate (Na_3_Ct·2H_2_O), tetrabutylammonium nitrate (TBA^+^NO_3_^−^), potassium hexacyanoferrate(III) (K_3_[Fe(CN)_6_]), chloride hexahydrate (FeCl_3_·6H_2_O), potassium hexacyanoferrate(II) trihydrate (K_4_[Fe(CN)_6_]·3H_2_O), crystal violet (CV), adenine, melamine, dopamine hydrochloride, thiram, aniline, nicotine, 2‐naphthalenethiol (2‐NT), 4‐Mercaptobenzoic acid (4‐MBA), hydrochloride, dichloromethane, chloroform, albumin, horse serum were purchased from Aldrich Ltd. Panobinostat was purchased from Selleck Chemicals. Silica nanospheres (SiO_2_, 50 nm, 10 mg mL‐1) were purchased from nanoComposix. All chemicals were used as received without further purification. Distilled deionized (DDI) water with a low resistivity of 18.2 MΩ was used for all experiments. Artificial serum samples were obtained by dissolving albumin in DDI water to form a 3.3 wt% solution.

### Colloid synthesis

Citrate‐reduced Au colloid was synthesized following a previously reported method.^[^ ^51^ ^]^ Briefly, 0.05 g of HAuCl_4_·3H_2_O was dissolved in 50 mL DDI in a frosted Erlenmeyer flask and heated with vigorous stirring until boiling under reflux. Then, 5.6 mL of aqueous sodium citrate (1 wt.%) was added to the precursor solution all at once. The boiling colloid was left to age under stirring for a total of 15 minutes after the addition of sodium citrate before being cooled down naturally to room temperature. The product colloid could be stored for at least 3 months at 4 °C and was used directly without any additional treatment.

Au@PB colloid was synthesized following a previously reported method with slight modifications.^[^ ^31^ ^]^ Briefly, an aqueous solution of K_3_[Fe(CN)_6_] (50 µL, 0.5 mM) was added to 5 mL of Au colloid under vigorous stirring and allowed to react for 5 minutes. Subsequently, 50 µL of aqueous solutions of K_4_[Fe(CN)_6_·3H_2_O] (5 mM) and FeCl_3_·6H_2_O (5 mM) were added simultaneously in a drop‐by‐drop fashion to the above Au colloid. The solution mixture was allowed to react under stirring for 3 hours, which led to the formation of a purple colloid. The product colloid was washed twice by centrifugation at 3000 rpm for 30 min and redispersed in DDI water. The final colloid was stored at 4 °C and used within one week.

### Synthesis of MWCNT‐Au@PB Pickering emulsions through promoter assisted co‐assembly

MWCNT‐cyclohexane dispersion was obtained by adding 2 mg of MWCNT powder into 20 mL of cyclohexane and sonicating using a Soniprep 150 Ultrasonic Disintegrator with 65 watts power for 30 minutes. For self‐assembly, 1 mL of the MWCNT‐cyclohexane dispersion was extracted and diluted by 2× with cyclohexane to be used as the oil phase. The diluted MWCNT cyclohexane solution was shaken vigorously with 500 µL of Au or Au@PB for 30 seconds with 50 µL of 1 mM TBA^+^NO_3_^−^ solution, which led to the formation of Pickering emulsions.

### Synthesis of SiO_2_‐Au@PB Pickering emulsions through promoter assisted co‐assembly

SiO_2_ stabilized Au@PB oil‐in‐water emulsions were prepared by vigorously shaking 0.5 mL of commercial SiO_2_ colloid with 1 mL of Au@PB colloid, 6.5 mL of DDI, 2.5 mL of cyclohexane solution, and 100 µL of 1 mM TBA^+^NO_3_^−^ (aq.) for 30 s.

### SERS using MWCNT‐Au@PB Pickering emulsion

SERS detection was carried out on a WITec Alpha 300 R Confocal Raman Microscope equipped with a 785 nm laser (using a 10× lens and 60 mW laser power, with an accumulation time of 30 seconds). Freshly prepared Pickering emulsions were used each time. To prepare the SERS samples, 50 µL of aqueous analyte solution (or 20 µL of analyte dispersed in oil) were introduced into the emulsion, followed by vigorous shaking of the emulsion sample for 10 seconds to promote adsorption. To carry out SERS measurements, an appropriate volume of the sample‐treated Pickering emulsion was poured into a hydrophobic polymer container, and the laser was focused onto the surface of a Pickering emulsion droplet to collect SERS signals. All spectra presented in this study were the result of averaging from at least 3 independent measurements.

All analyte concentrations mentioned were the final concentration of the analyte molecule within the emulsion sample, which is 10× lower than the concentration of the analyte molecule in the initial sample.

### Microscopy characterizations

Optical microscopy of the Pickering emulsions was performed using a Nikon SMZ800 microscope. Scanning electron microscopy‐energy‐dispersive X‐ray spectroscopy (SEM‐EDX) analysis was carried out using a Quanta FEG 250 instrument, operating at an acceleration voltage of 30 kV within a high vacuum environment (8 × 10^−5^ mbar). To prepare the SEM samples, 100–200 µL of ethanol was introduced into the Pickering emulsion samples to de‐emulsify the emulsions and generate interfacial arrays. These arrays were subsequently dried onto aluminum foil in a vacuum oven. Transmission electron microscopy (TEM) characterizations were conducted using a Joel JEM‐1400 Plus Transmission Electron Microscope and a TALOS F200X G2: Scanning/transmission electron microscope (S/TEM) equipped with EDX system for chemical analysis. The Au@PB colloid sample was prepared by directly drying a diluted droplet of the colloidal solution onto the carbon film (S160200 mesh Cu (25)). Pickering emulsion samples were prepared by extracting and depositing a single emulsion droplet onto the carbon film using a pipette, followed by drying at room temperature.

### Contact angle measurement

Three‐phase Contact angle measurements were conducted using a First Ten Angstroms FTA1000 goniometer. The samples were fabricated in a manner consistent with the preparation of SEM samples. Then, they were affixed at the bottom of a glass container and immersed in cyclohexane. Following immersion, a 3 µL droplet of water was dropped onto the sample's surface, and contact angle was measured.

## Conflict of Interest

The authors declare no conflict of interest.

## Author Contributions

Conceptualization: YX, SB; Methodology: YZ, YX, ZY, RC; Investigation: YZ, CL, YX, ZY, RC; Visualization: YX, CL, YZ, SB; Supervision: YX, CL, SB; Funding: SB, YX; Writing—original draft: CL, YZ, YX; Writing—review & editing: YX, ZY, CL

## Supporting information



Supporting Information

## Data Availability

The data that support the findings of this study are available in the supplementary material of this article.
