# Supplementary material for: Untying Surface Chemistry and Emulsion Stability to Construct Multifunctional Pickering Emulsion SERS Sensors for Pretreatment‐Free Quantitative Analysis in Bio‐Media
Source: Adv Sci (Weinh). 2025 May 14;12(28):2505714. doi: 10.1002/advs.202505714 (PMC12302603; doi:10.1002/advs.202505714)
Supplement: Supplementary file 1 — Supporting Information [file ADVS-12-2505714-s001.docx]

Supplementary information

Untying surface chemistry and emulsion stability to construct multifunctional Pickering emulsion SERS sensors for Pretreatment-Free Quantitative analysis in bio-media

*Yingrui Zhang^3^, Chunchun Li^2,3^*, Ruairi Carland^3^, Ziwei Ye^1^, Steven E. J. Bell^3^, and Yikai Xu^1^**

*^1^ Key Laboratory for Advanced Materials and Feringa Nobel Prize Scientist Joint Research Center, Frontiers Science Center for Materiobiology and Dynamic Chemistry, School of Chemistry and Molecular Engineering, East China University of Science and Technology, 130 Meilong Road, Shanghai 200237, China.*

*^2^ School of Materials Science and Engineering, East China University of Science and Technology, 130 Meilong Road, Shanghai 200237, China*

*^3^ School of Chemistry and Chemical Engineering, Queen’s University Belfast, University Road, Belfast, BT7 1NN, UK*

**
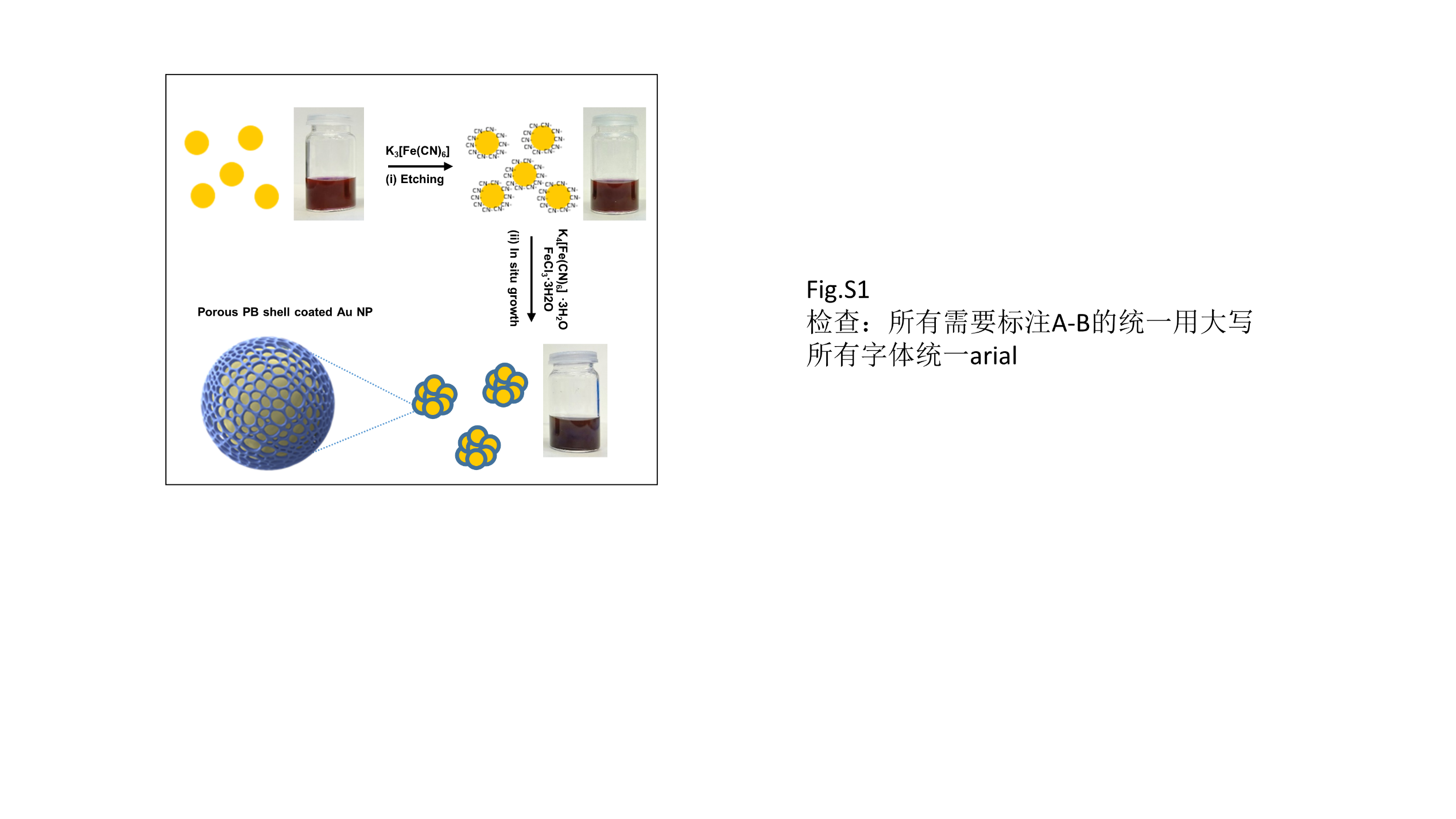
**

**Figure S1.** Schematic illustration and optical images of the synthesis process of Au@PB colloid.

The synthesis of Au@PB colloidal nanoparticles (NPs) follows a double-precursor process, which involves (i) an etching process on the Au NPs surface by [Fe(CN)_6_]^3-^ followed by (ii) the in-situ growth of a PB shell on the Au NPs surface with equal-molar amounts of Fe^3+^/Fe^2+^. The reactions took place in (ii) is shown as below:

(1) $3\mathrm{Fe}^{2+}+2\left[ \mathrm{Fe}\left( \mathrm{CN} \right)_{6} \right]^{3-}=\mathrm{Fe}_{3}{[\mathrm{Fe}{(CN)}_{6}]}_{2}\downarrow$

(2) $4\mathrm{Fe}^{3+}+3\left[ \mathrm{Fe}\left( \mathrm{CN} \right)_{6} \right]^{4-}=\mathrm{Fe}_{4}{[\mathrm{Fe}{(CN)}_{6}]}_{3}\downarrow$


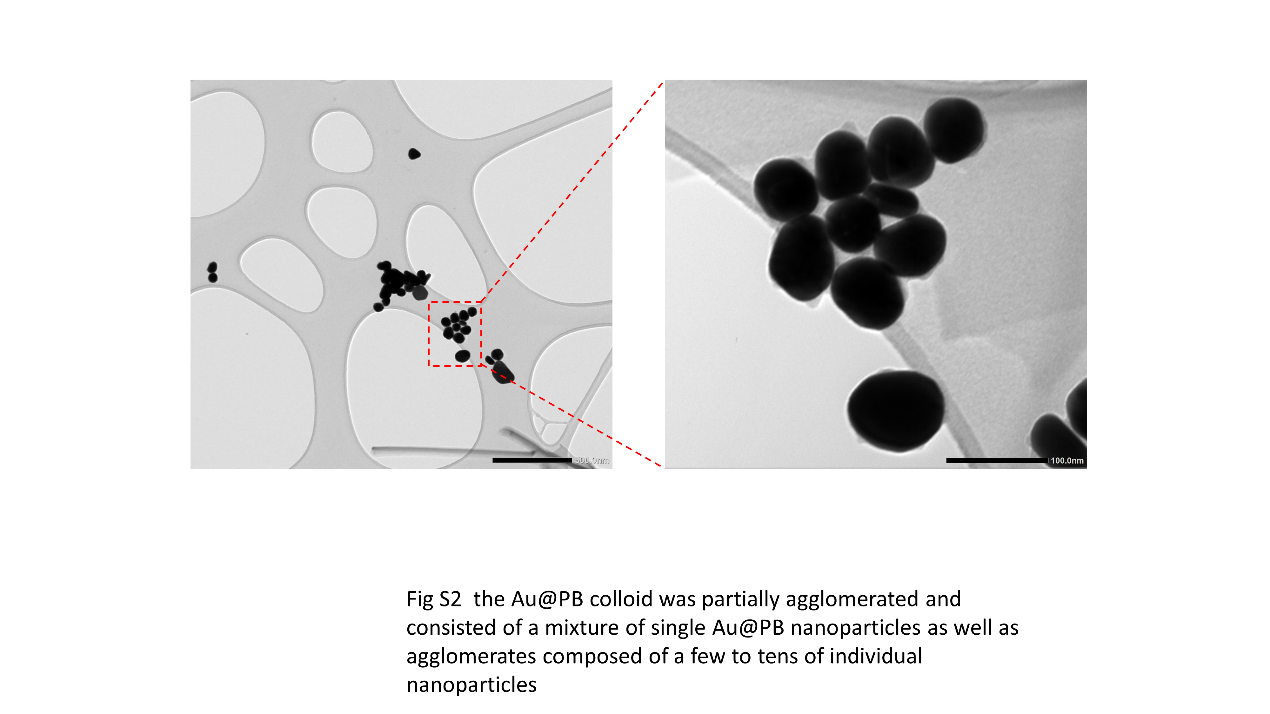


**Figure S2.** High and low magnification TEM images of a typical Au@PB colloid sample which was partially agglomerated.


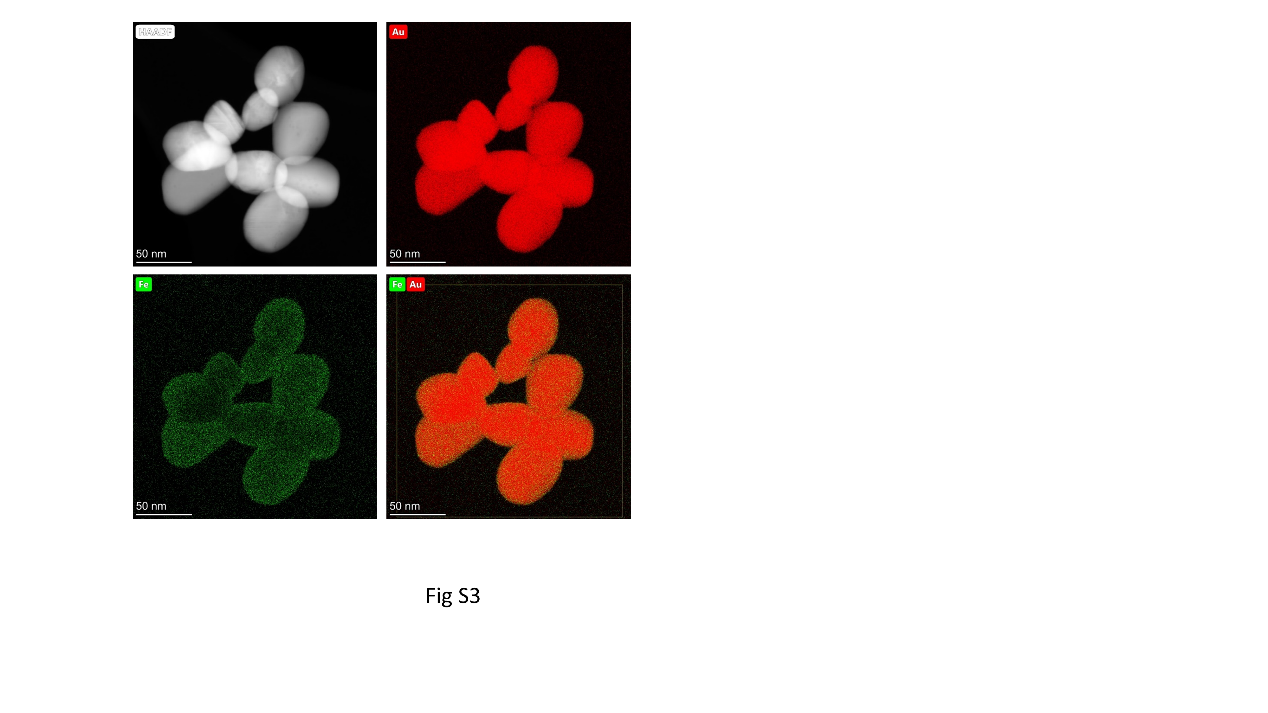


**Figure S3.** HAADF-STEM-EDX mapping of a typical agglomerate in the Au@PB colloid sample.

**Supplementary Table 1.** **The values of parameters** $\boldsymbol{p}$ **and** $\boldsymbol{z}$ **of Equation 2 (main text) for different cases.** The values are most accurate for calculating the maximum capillary pressure between densely packed solid spherical NP bilayers.

| Situation parameter | $\theta<90^{\circ}$ (o/w);  $\theta>90^{\circ}$ (w/o) | $90^{\circ}\leq\theta\leq129.3^{\circ}$ (o/w);  $50.7^{\circ}\leq\theta\leq90^{\circ}$ (w/o) |
| --- | --- | --- |
| $p$ | 4.27 | 2.73 |
| $z$ | 0.405 | 0.633 |

**
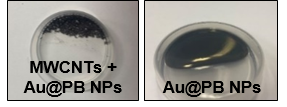
**

**Figure S4.** Optical images of the Au@PB assemblies and MWCNT-Au@PB emulsions.


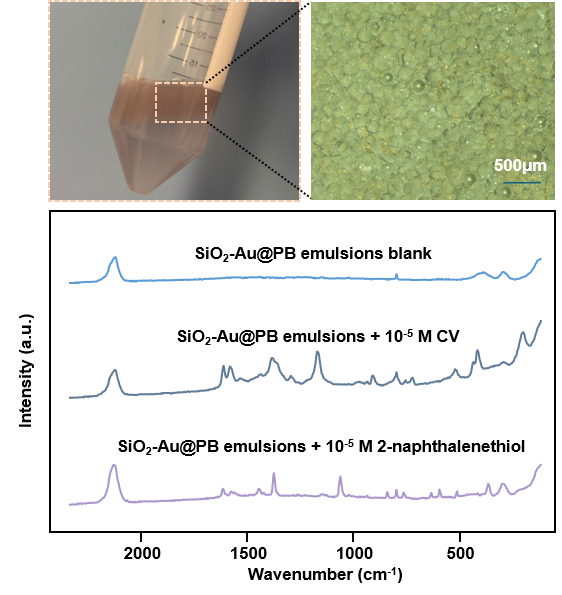


**Figure S5.** Optical images of Pickering emulsions formed with SiO_2_ and Au@PB nanoparticles and their SERS activity investigated using CV and 2-naphthalenethiol as analytes.


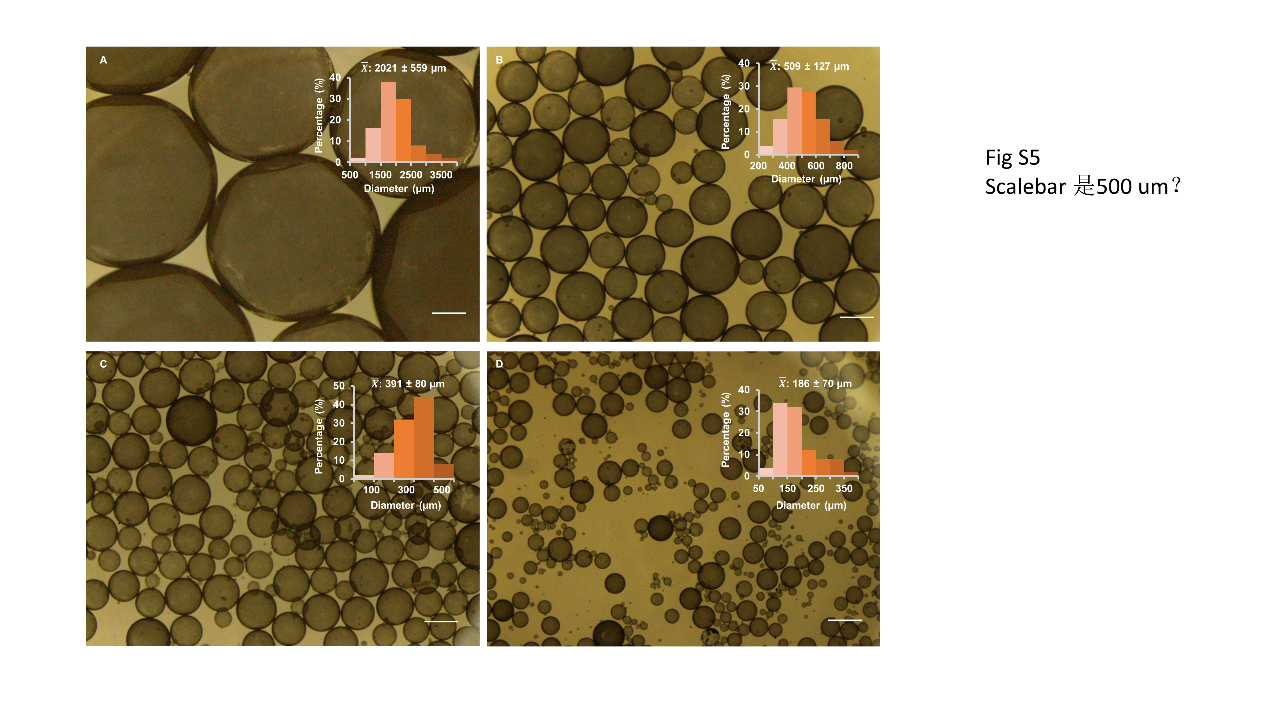


**Figure S6.** The average size of MWCNT-Au@PB emulsions at different MWCNT concentrations. Optical microscopy images and size distribution charts showing the average size of MWCNT-Au@PB Pickering emulsions formed with (A) 0.025 (B) 0.1 (C) 0.15 (D) 0.2 mg/mL of MWCNTs, respectively. The scale bars correspond to 500 µm.


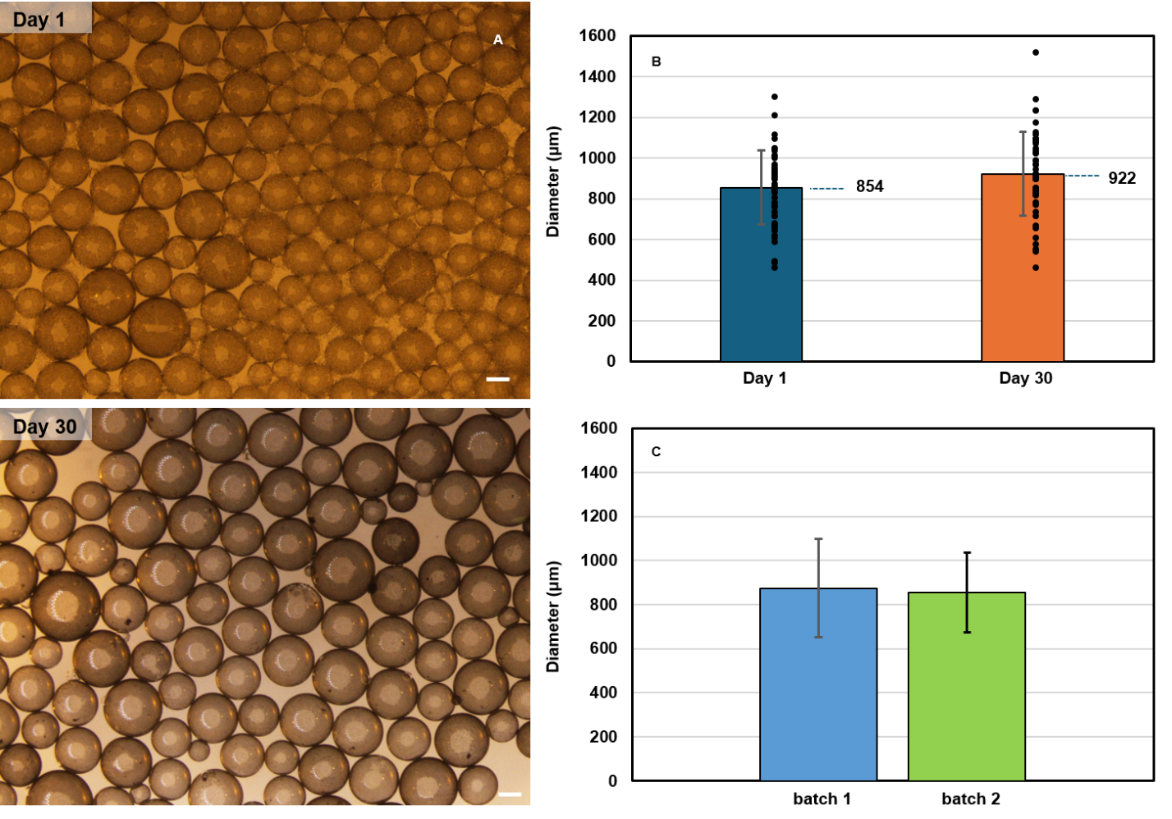


**Figure S7.** (A-B) Optical microscopy images and corresponding diameter chart showing the average size of a freshly prepared MWCNT-Au@PB Pickering emulsion sample and the same sample measured after 30 days. The scale bars correspond to 500 µm. (C) Diameter chart showing the average size of 2 different batch of MWCNT-Au@PB Pickering emulsion sample.


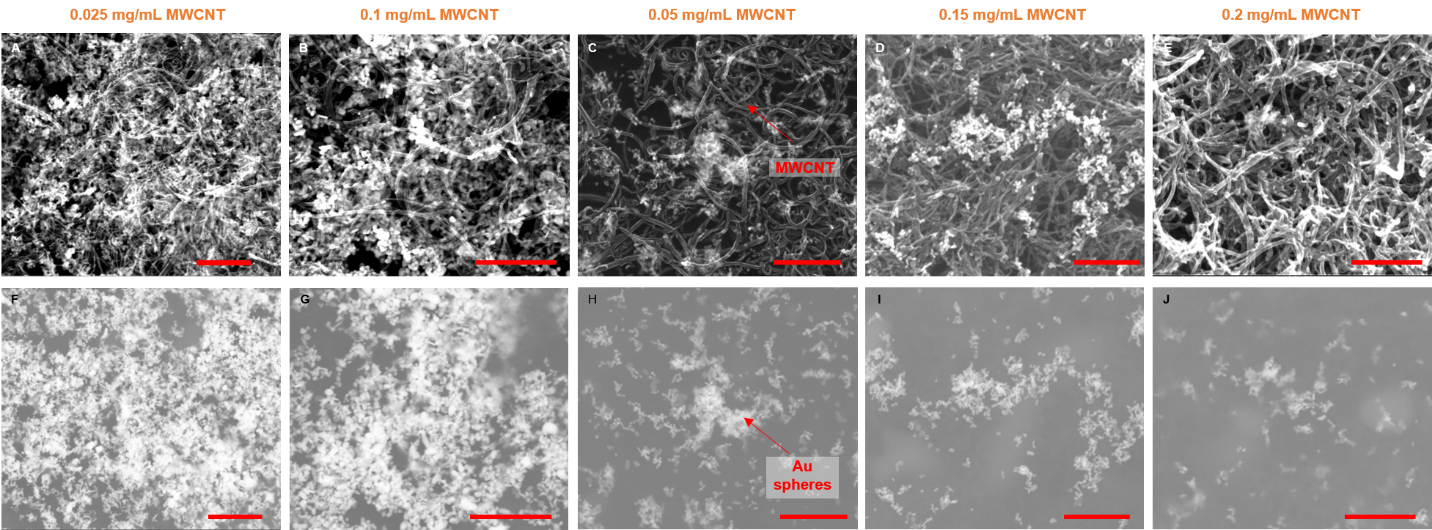


**Figure S8.** Scanning electron microscopy images of the mixed nanoparticle layer in MWCNT-Au@PB emulsions stabilized with different concentrations of MWCNTs imaged (A-E) using a secondary detector and (F-J) using a backscattering detector. The scale bars correspond to 1 µm.


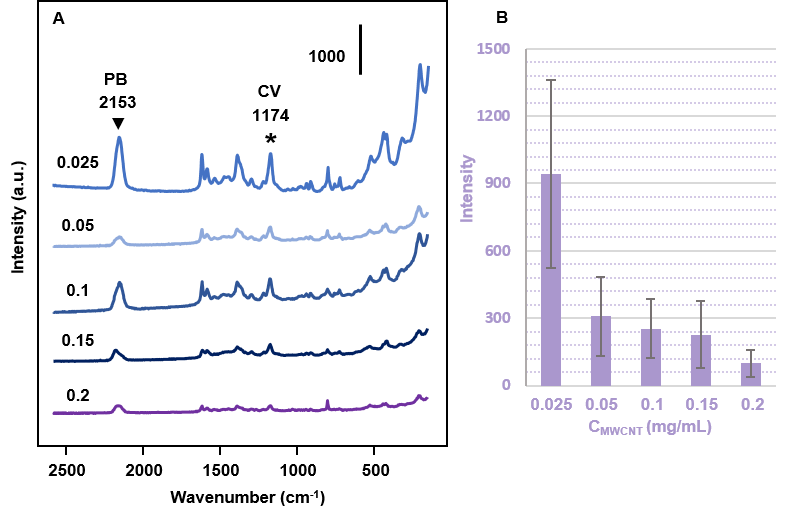


**Figure S9.** (A) Typical SERS spectra obtained from MWCNT-Au@PB emulsions formed with different concentrations of MWCNT stabilizers treated with 10^-5^ M of CV. (B) Plot showing the average SERS signal intensity of 10^-5^ M of CV obtained with MWCNT-Au@PB emulsions formed with different concentrations of MWCNT stabilizers.


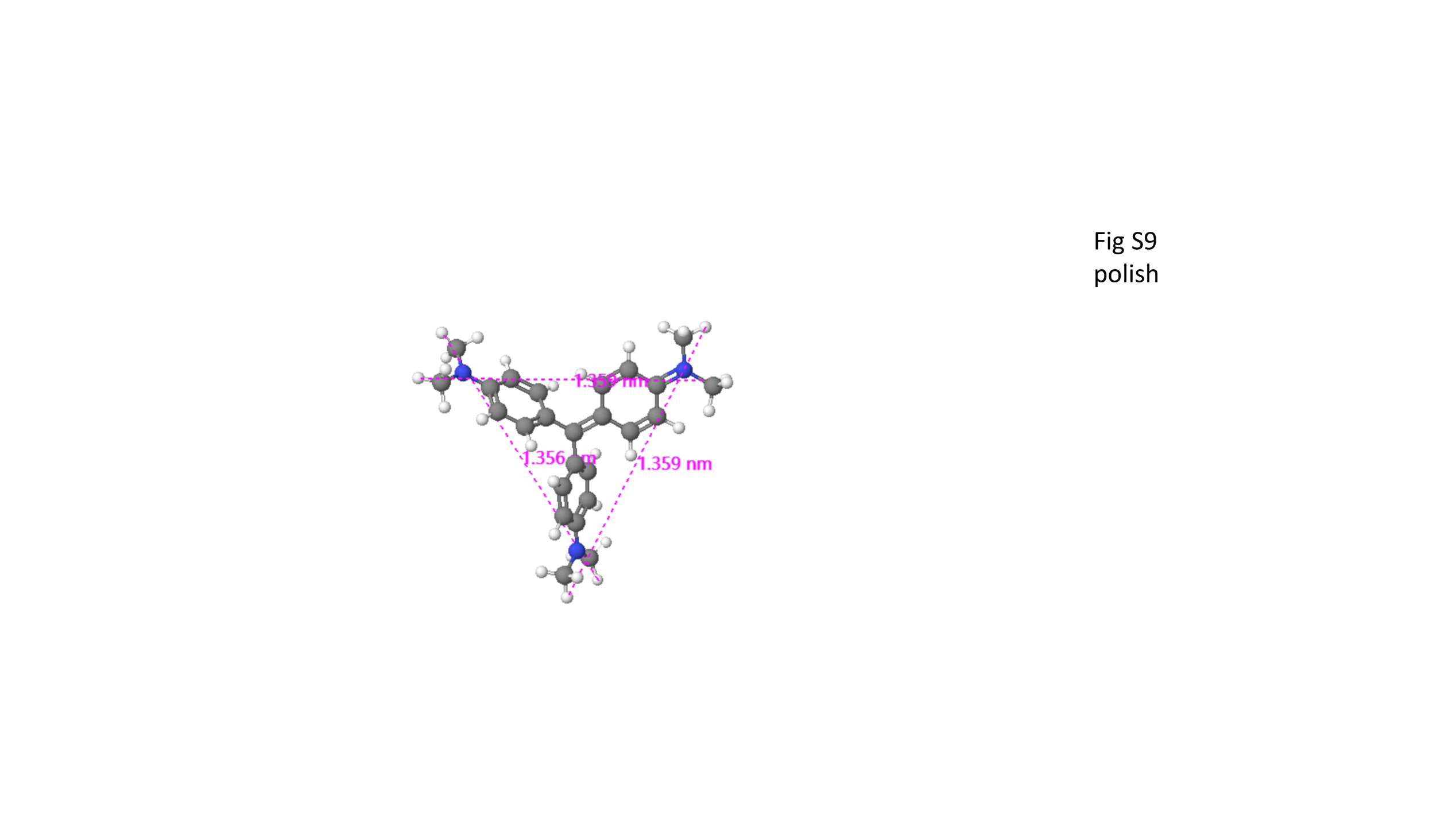


**Figure S10.** Chemical structure and predicted molecular diameter of crystal violet.


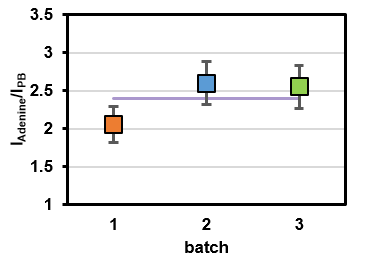


**Figure S11.** Plot showing the value of I_Adenine_/I_PB_ of 3 different batches of MWCNT-Au@PB emulsions. The purple line represents for the average value of the 3 batches.


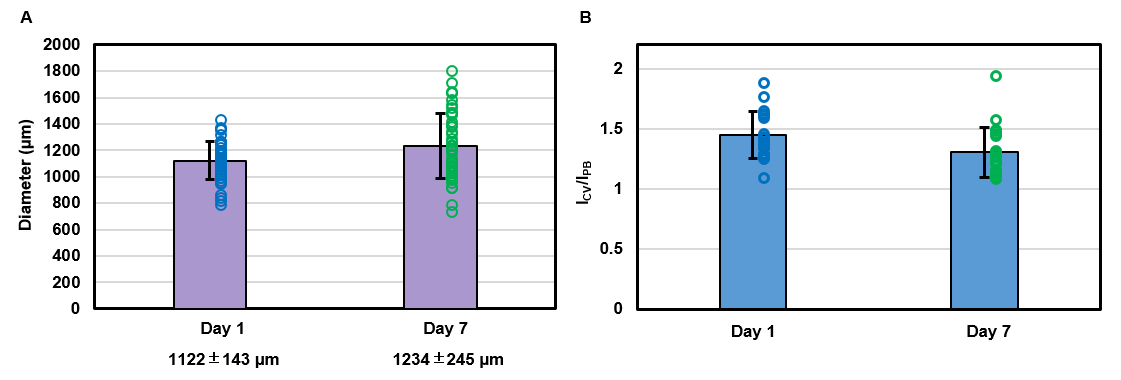


**Figure S12.** Histogram showing the stability of emulsion droplets in the presence of 5 x 10^-5^ M CV.

**Table S2.** **Table comparing previous SERS studies performed with Pickering emulsions as the enhancing substrate versus the current work.**

| **Plasmonic component** | **Modifier ligand** | **Target analyte** | **Ref** |
| --- | --- | --- | --- |
| Ag nanocubes | perfluorodecanethiol | methylene blue;  rhodamine 6G;  malachite green;  dimethyl yellow | (*21*) |
| Ag nanoparticles | FC143 surfactant | FC143 surfactant | (*20*) |
| Au nanostars | perfluorodecanethiol | methylene blue;  rhodamine 6G;  crystal violet;  DNA nucleobases;  canonical deoxyribonucleoside monophosphates | (22) |
| Ag nanoparticles | HS-β-cyclodextrin | o-phenylenediamine;  benzotriazole;  2,3-diaminophenazine | (*23*) |
| Ag and Au nanoparticles | perfluorodecanethiol | Bacterial strains | (*24*) |
| Au@PB nanoparticles | / | crystal violet;  adenine;  aniline;  dopamine;  melamine;  thiram;  panobinostat;  2-naphthalenethiol;  4-mercaptobenzoic acid;  nicotine | This work |


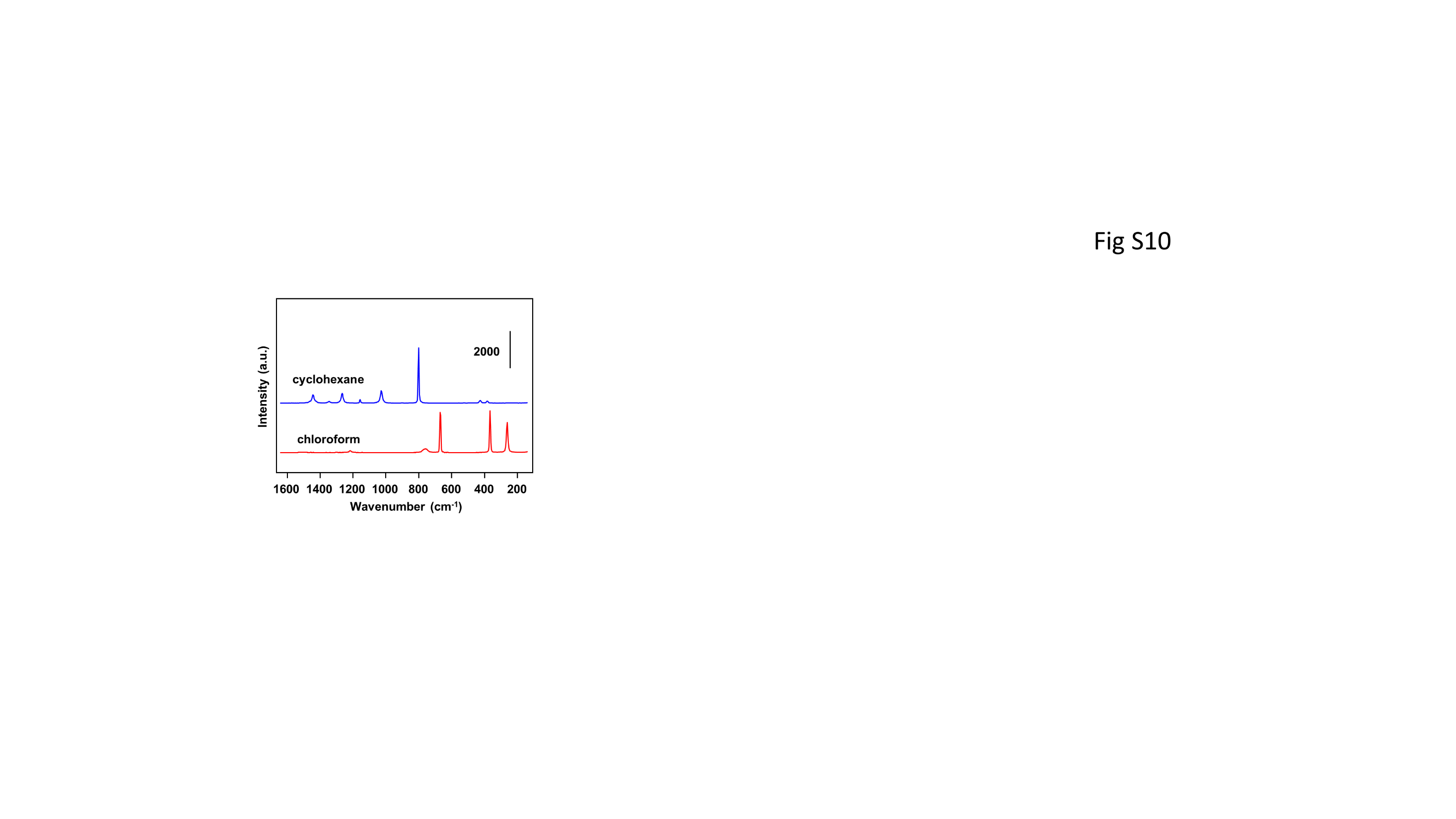


**Figure S13.** Raman spectra of pure cyclohexane and chloroform.


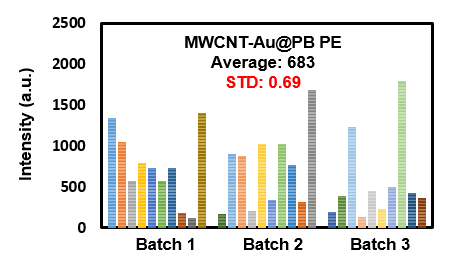


**Figure S14.** Plot showing the SERS signal intensity of adenine measured from 30 randomly selected points on 30 different MWCNT-Au@PB emulsion droplets obtained from 3 batches of emulsion samples. The final concentration of adenine was 10^-5^ M.


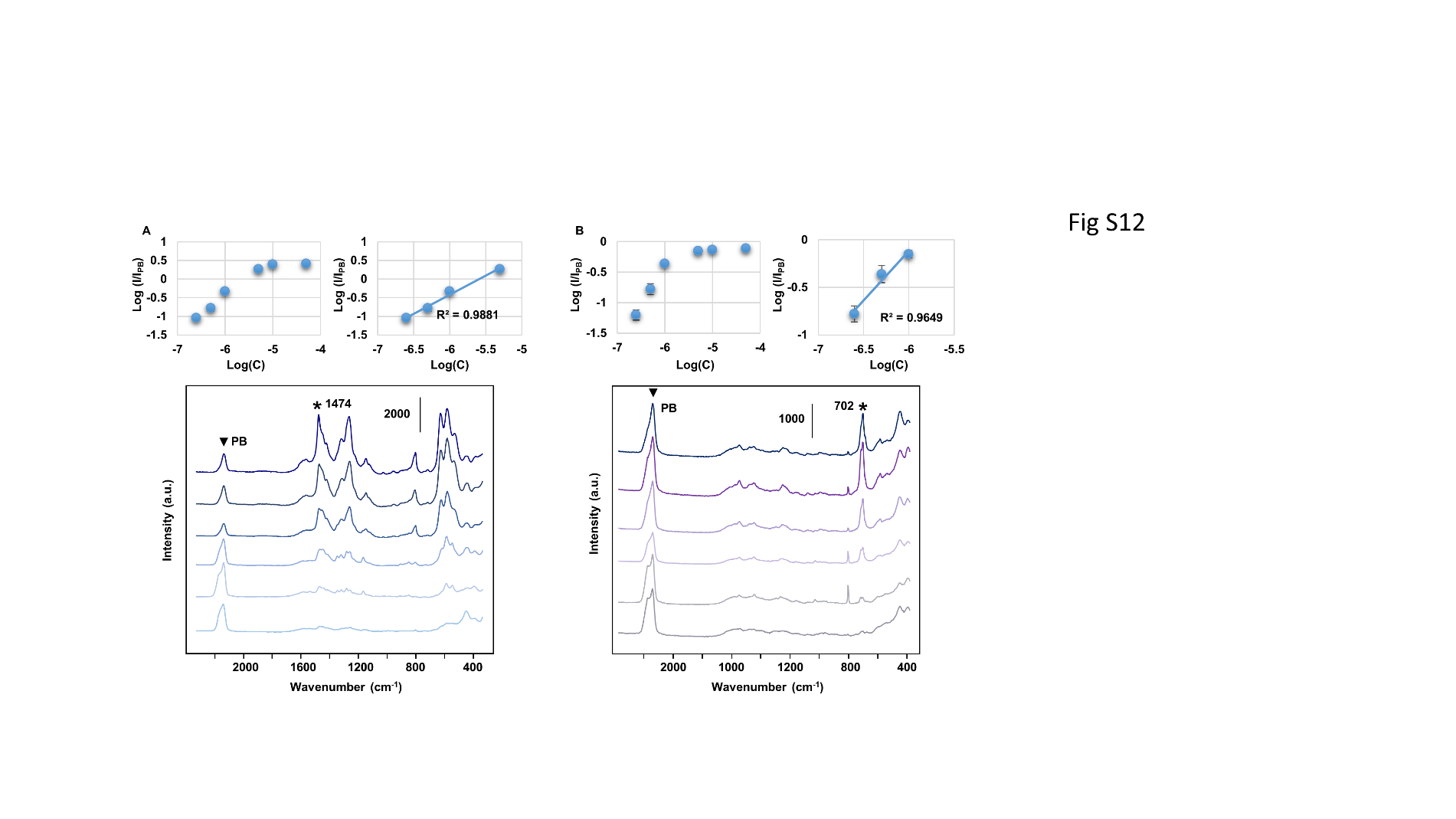


**Figure S15.** (A) SERS spectra and corresponding calibration curve (using signal of PB as the internal standard) of various concentrations of dopamine in artificial serum obtained using MWCNT-Au@PB emulsions. The characteristic peaks selected for dopamine was centred at 1474 cm^-1^. (B) SERS spectra and corresponding quantitative curve (using signal of PB as the internal standard) of various concentrations of melamine in artificial serum obtained using MWCNT-Au@PB emulsions. The characteristic peaks selected for melamine was centred at 702 cm^-1^.


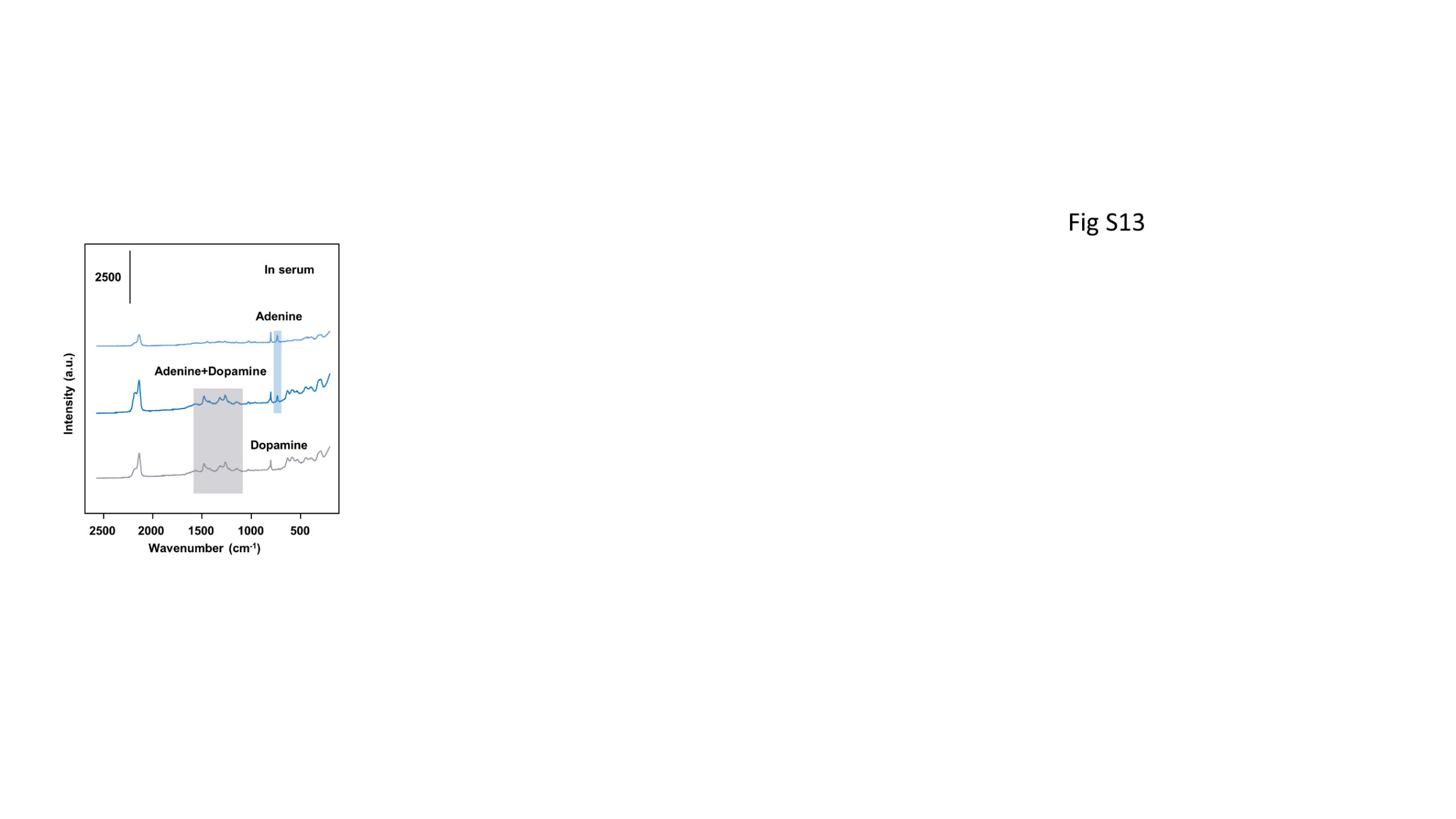


**Figure S16.** SERS spectra of 10^-5^ M adenine, dopamine and their mixture in horse serum.
